# Keratinocyte growth factor-2 intratracheal instillation significantly attenuates ventilator-induced lung injury in rats

**DOI:** 10.1111/jcmm.12269

**Published:** 2014-03-21

**Authors:** Jing Bi, Lin Tong, Xiaodan Zhu, Dong Yang, Chunxue Bai, Yuanlin Song, Jun She

**Affiliations:** Department of Pulmonary Medicine, Zhongshan Hospital, Fudan UniversityShanghai, China

**Keywords:** keratinocyte growth factor-2, mechanical ventilation, alveolar type II cell, surfactant protein, inflammatory cytokines

## Abstract

Preservation or restoration of normal alveolar epithelial barrier function is crucial for pulmonary oedema resolution. Keratinocyte growth factor-2 (KGF-2), a potent epithelial cell mitogen, may have a role in preventing ventilator-induced lung injury (VILI), which occurs frequently in mechanically ventilated patients. The aim of the study was to test the role of KGF-2 in VILI in rats. Forty healthy adult male Sprague-Dawley rats were randomly allocated into four groups, where rats in Groups HVZP (high-volume zero positive end-expiratory pressure) and HVZP+KGF-2 were given intratracheally equal PBS and 5 mg/kg KGF-2 72 hrs before 4 hrs HVZP ventilation (20 ml/kg), respectively, while PBS and KGF-2 were administered in the same manner in Groups Control and KGF-2, which underwent tracheotomy only with spontaneous breathing. Inflammatory cytokines (tumour necrosis factor-α, macrophage inflammatory protein 2), neutrophil and total protein levels in bronchoalveolar lavage fluid and surfactant protein mRNA expression in lung tissue were detected; the number of alveolar type II cells, lung water content and lung morphology were also evaluated. The results indicate that pre-treatment with KGF-2 showed dramatic improvement in lung oedema and inflammation compared with HVZP alone, together with increased surfactant protein mRNA and alveolar type II cells. Our results suggest that KGF-2 might be considered a promising prevention for human VILI or other acute lung injury diseases.

## Introduction

Mechanical ventilation is the main life-sustaining tool in acute lung injury (ALI)/acute respiratory distress syndrome (ARDS), but unequivocal evidence from both experimental and clinical researches has suggested that mechanical ventilation could aggravate or even initiate lung injury, which is known as ventilation-induced lung injury (VILI) [1]. Even low-tidal volume strategies may cause the undesirable side effects of cyclic hyperinflation of some lung areas [2]. Ventilator-induced lung injury is characterized by increased endothelial and epithelial permeability and inflammatory processes [3]. Therefore, ideal VILI treatment or prophylaxis should protect the integrity of alveolar-capillary membrane and reduce inflammatory processes. However, currently, no effective pharmacotherapies are available to prevent or attenuate VILI.

Keratinocyte growth factor-2 (KGF-2), namely fibroblast growth factor-10 (FGF-10), has been reported to mediate epithelial–mesenchymal interactions, which is essential for lung development [4–10], and recently it has been found that it may be implicated in preventing lung injury from various stresses [11–13]. With similarities to KGF, KGF-2 is a 20-kD heparin-binding protein predominantly expressed by mesenchymal cells. It binds with high affinity to spliced variants of FGF receptor 2-IIIb (FGFR2-IIIb) and FGFR1III-b that are expressed by epithelial and endothelial cells. Otherwise, unlike KGF, KGF-2 also binds to FGFR1III-b [7,14–17], which may explain the severely impaired lung development of FGF-10 null mice. The cooperation of FGFR1IIIb and FGFR2IIIb plays an important role in maintaining epithelial and endothelial barrier functions [18,19]. One of our recent studies has discovered the protective effects of KGF-2 on endothelial cells in a high-altitude pulmonary oedema model [20]. In addition, unlike KGF, KGF-2 has been demonstrating its safety by using phase II trials for the systemic, injectable formulation in ulcerative colitis and in the prevention of mucositis [21].

This is the first study to estimate the KGF-2 effect on VILI animal model. Considering the high safety and different pharmacokinetic/pharmacodynamics (PK/PD) of KGF-2 compared with KGF, it is important to study the effects and administration approaches of KGF-2 in acute lung injury induced by high-volume zero positive end-expiratory pressure (PEEP) (HVZP) in order to provide laboratories evidence for potential clinical implication.

## Materials and methods

### Animals

Forty healthy Specific pathogen Free Male Sprague-Dawley (SD) rats (the Animal Center of Fudan University, Shanghai, China) weighing 200–250 g were used. Rats were maintained in the laboratory animal centre of Zhongshan Hospital, Fudan University, Shanghai with clean, controlled temperature and independent ventilation environment. The animals had free access to food and water, but food was withdrawn 12 hrs before experiment. All procedures were approved by the Committee of Animal Care of Fudan University. All animals were handled in accordance with the Guide for the Care and Use of Laboratory Animals published by the National Institutes of Health, and efforts were made to minimize suffering and number in our study.

### Experimental groups

Animals were randomly assigned to the following experimental groups (*n* = 10 in each group): (*i*) Control group: underwent tracheotomy only with spontaneous breathing, PBS-treated before tracheotomy; (*ii*) KGF-2 group: KGF-2-treated before tracheotomy; (*iii*) HVZP group: underwent high-tidal volume ventilation strategy for 4 hrs, PBS-treated 72 hrs before ventilation; (*iv*) HVZP+ KGF-2 group: KGF-2-treated 72 hrs before ventilation.

### KGF-2 intratracheal instillation

Firstly, rats were anesthetized by intraperitoneal injection of 10% chloral hydrate (3.33 ml/kg), and then either rhKGF-2 (recombinant human KGF-2, 5 mg/kg in 0.5 ml; New Summit Biopharma, Shanghai, China) or equal PBS (pH 7.2) was intratracheal instillation directly 72 hrs before ventilation challenge with an 18-gauge needle followed by an injection of 0.5 ml air. Finally, animals were allowed to recover for 15 min.

### Ventilator-induced lung injury in rat model

The protocol was performed as described previously [22]. Briefly, rats were anaesthetized by intraperitoneal injection of urethane (1.5 g/kg; Sigma-Aldrich, St Louis, MO, USA). They were then placed in a supine position and with a rectal temperature probe inserted for temperature measurement. Throughout the experiment, rat body temperature was monitored and maintained at 37°C by using a heating lamp. Anterior neck soft tissue was dissected after sterilization to expose the trachea. The tracheostomy was then performed and a 14-gauge intravenous (i.v.) catheter (Terumo, Tokyo, Japan) was inserted as a tracheostomy tube. Left cervical artery was cannulated for blood gas analysis by using PE-50 tubing. Each animal was injected with vecuronium bromide (1 mg/kg; N.V. Organon, Oss, The Netherlands) intraperitoneally to facilitate mechanical ventilation, after which rats were ventilated with room air at 85 breaths/min for 4 hrs with V_T_ of 20 ml/kg and zero end-expiratory pressure with a rodent volume ventilator (Harvard Rodent Ventilator 683, South Natick, MA, USA). Compared with experimental group, rats in the control group also underwent tracheotomy only without actual ventilation. Rats were allowed to adapt to the surgery stress for at least 30 min. before the experiment.

### Arterial blood gas

At the end of each experiment, blood (0.5 ml) was obtained from the left cervical artery and arterial blood gas levels were immediately measured with a blood gas analyzer (Medica Easy Blood Gas, Medica, Bedford, MA, USA).

### Lung wet-to-dry weight ratio (W/D)

The rats were killed after 4 hrs of ventilation. The trachea was then clamped at end-inspiration (inflated at 8 ml/kg) before sternotomy. The gross vessels were cut and blood passively emptied. The middle lobe of right lung was excised and weighed for determination of the final wet lung weight. Additionally, dry weight was measured by placing the middle lobe of right lung in an oven at 60°C for 5 days. The W/D weight ratio was then calculated. Meanwhile, the lower lobe of right lung was filled with 10% buffered formalin for histological analysis. Other lobe of right lung was frozen for further biochemical assays.

### Bronchoalveolar lavage

Bronchoalveolar lavage (BAL) was selectively performed with the left lung. The left lung was slowly infused with 2 ml PBS (4°C) and washed for two to three times (over 30 sec.) to get bronchoalveolar lavage fluid (BALF). Generally, more than 90% of infused fluid was recovered.

#### Cell counting

Bronchoalveolar lavage fluid samples were centrifuged at 132 *g* for 10 min. at 4°C, and then the supernatant was removed and stored at −80°C. The pellet was resuspended with 0.5 ml of PBS. The total number of nucleated cells in BALF was counted with a haemocytometer. Total resuspended BAL cells were centrifuged (298 *g* for 15 min., LTP-A Cytocentrifuge, Experimental Instrument Factory of Military Medical Sciences, Beijing, China), transferred to slides and stained with Wright's-Giemsa (Jiancheng, Nanjing, China). Finally, the slides were quantified for polymorphonuclear leucocytes by counting a total of 200 cells/slide at ×40 magnification.

#### BAL protein concentration

Bronchoalveolar lavage protein concentration was measured by using a bicinchoninic acid protein assay kit according to the manufacturer's instruction (Thermo Scientific, Pittsburgh, PA, USA).

#### Cytokines in BAL

Tumour necrosis factor-α (TNF-α) and macrophage inflammatory protein (MIP)-2 in BAL were measured by using a rat TNF-α ELISA kit (ebioscience, San Diego, CA, USA) and a pre-coated rat MIP-2 colorimetric sandwich ELISA kit (R&D Systems, Minneapolis, MN, USA) according to the manufacturers’ recommendations respectively.

### Lung homogenate myeloperoxidase activity

The activity of lung homogenate myeloperoxidase (MPO) was used as a marker of pulmonary neutrophil infiltration and was examined by using the assay kit (Jiancheng) according to the manufacturer's protocol.

### Lung morphology

#### Optical microscope

Immediately after the BAL, the right lower lobe was removed and fixed in 10% formaldehyde, then embedded in paraffin, stained with haematoxylin and eosin, and examined by a pathologist who was blinded to the experimental protocol. Lung injury was scored according to the following items: (*i*) alveolar congestion, (*ii*) haemorrhage, (*iii*) infiltration of neutrophils into the airspace or the vessel wall, and (*iv*) thickness of the alveolar wall. Each item was graded according to a five-point scale: 0, minimal (little) damage; 1, mild damage; 2, moderate damage; 3, severe damage; and 4, maximal damage.

#### Transmission electron microscopy (TEM)

The lungs were inflated with fixative (2.5% glutaraldehyde, 50 mM sodium cacodylate buffer) *in situ* to a constant pressure of 25–30 cm and removed into fixative and stored at 4°C for a minimum of 24 hrs. Samples of parenchyma of the left and right upper lobe were post-fixed in 1% osmium tetroxide in 50 mM sodium cacodylate buffer, dehydrated in graded alcohol and propylene oxide, and subsequently embedded in Araldite epoxy resin. Initially semithin sections (1 μm) were cut by using a Reichart Ultracut E ultramicrotome and stained with 1% toluidine blue (in 1% sodium tetraborate) for the purposes of orientation and initial morphological assessment. Ultrathin sections (80–100 nm) were cut, contrasted with uranyl acetate and lead citrate, and examined with a Hitachi 7000 TEM.

### Quantitative real-time polymerase chain reaction

The mRNA level of surfactant protein A (SP-A), surfactant protein B (SP-B) and surfactant protein C (SP-C) in lung tissue were measured by using quantitative real-time polymerase chain reaction (qRT-PCR).

### Immunohistochemistry for SP-C

Surfactant protein-C is only synthesized in the alveolar type II cells [23]. Thus, the numbers of alveolar type II cells were measured by using immunostaining with anti-SP-C antibody. Immunoreactive alveolar type II cells were identified as cells with brown staining within the cytoplasm and quantified by counting 10 randomly selected fields of view per slide (×400 magnification, 1 slide/animal). All slides were evaluated in a blinded fashion.

### Statistical analysis

Data were presented as mean ± SD and analysed by using SPSS version 11.5 statistical software (SPSS, Chicago, IL, USA). Comparisons between multiple groups were performed by one-way anova procedures, followed by the Bonferroni *post hoc* test for intergroup comparisons. The histological semiquantitative analysis was compared by the non-parametric Mann–Whitney test. A *P* value < 0.05 was considered significant.

## Results

### KGF-2 improves HVZP-induced hypoxaemia and protein-rich oedema

In this study, the group assignment was based on the following rational: KGF-2 other than KGF was used in this study because of proved relative safety of KGF-2 in phase II clinical trials [21]. The dose and administration time of KGF-2 were selected based on results from our preliminary study (1, 2, 5 and 10 mg/kg has been tested with 5 mg/kg providing maximum protection, on the other hand, KGF-2 showed maximum lung protection 3 days after KGF-2 pre-treatment; detailed in Data S1).

**Figure 1:**
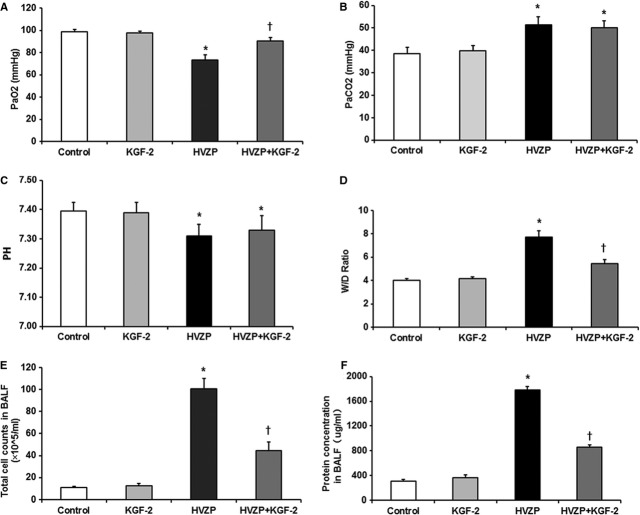
KGF-2 improves HVZP-induced hypoxaemia and protein-rich oedema. (A) Partial pressure of oxygen (PaO_2_) after 4 hrs mechanical ventilation. PaO_2_ in HVZP group was significantly lower than that in the control group (*P* < 0.05). KGF-2 pre-treatment significantly increased PaO_2_ compared with the HVZP groups (*P* < 0.05). (B) PaCO_2_ after 4 hrs mechanical ventilation. PaCO_2_ in HVZP group was significantly higher than that in the control group (*P* < 0.05). No statistical difference was discovered between HVZP group and HVZP+KGF-2 group. (C) pH value after 4 hrs mechanical ventilation. pH value in HVZP group was significantly lower than that in the control group (*P* < 0.05). No statistical difference was discovered between HVZP group and HVZP+KGF-2 group. (D) Lung W/D weight ratios. W/D ratio in HVZP group was significantly higher than that in control group (*P* < 0.05) and HVZP+KGF-2 group (*P* < 0.05). (E) The number of total cells recovered in the bronchoalveolar lavage fluid (BALF). Total Cell Count in HVZP group was significantly higher than that in control group (*P* < 0.05) and HVZP+KGF-2 group (*P* < 0.05). (F) The total protein contents recovered in the bronchoalveolar lavage fluid. Total protein contents in HVZP group were significantly higher than those in control group (*P* < 0.05) and HVZP+KGF-2 group (*P* < 0.05). No statistical difference was discovered between KGF-2 and control groups. *P < 0.05 *versus* control group; †*P* < 0.05 *versus*HVZP group.

After 4 hrs MV, the partial pressure of oxygen (PaO_2_) of rats in HVZP group was significantly lower than that in control group (*P* < 0.05, Fig.1A). KGF-2 pre-treatment significantly increased PaO_2_ compared with the HVZP groups (*P* < 0.05, Fig.1A). In addition, PH value was significantly lower and the partial pressure of carbon dioxide (PaCO_2_) was significantly higher in animals receiving high V_T_ (*P* < 0.05, Fig.1B and C), but values were similar between those pre-treated with KGF-2 and PBS.

A significant increase in W/D was observed in HVZP group compared with the controls (*P* < 0.05, Fig.1D). Keratinocyte growth factor-2 pre-treatment significantly decreased lung water compared with the HVZP group (*P* < 0.05, Fig.1D). The number of total cells and total protein contents recovered in BALF were also measured to evaluate lung oedema. Finally, we discovered that rats treated with HVZP ventilation showed significantly higher total cell counts and total protein concentration in BALF than the control group (*P* < 0.05, Fig.1E and F). Keratinocyte growth factor-2 pre-treatment significantly decreased total cell counts and total protein concentration in BALF than the HVZP group (*P* < 0.05, Fig.1E and F).

### KGF-2 reduces inflammatory processes in VILI

**Figure 2:**
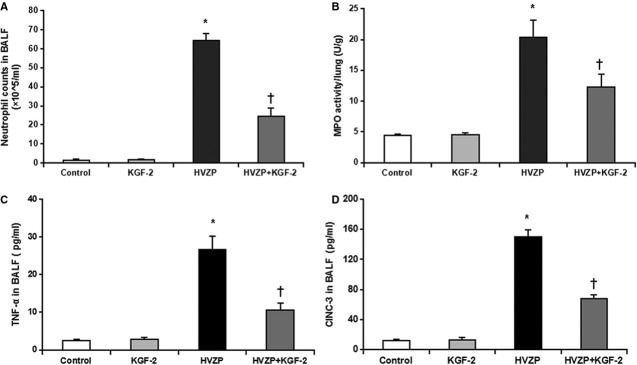
KGF-2 reduces neutrophil infiltration and inflammatory cytokines in the BALF. (A) The number of neutrophil in the bronchoalveolar lavage fluid (BALF). The number of neutrophil in the BALF in HVZP group was significantly higher than those in the control group (*P* < 0.05). KGF-2 pre-treatment significantly decreased the number of neutrophil in the BALF compared with the HVZP group (*P* < 0.05). (B) Lung myeloperoxidase (MPO) activity. MPO activity in HVZP group was significantly higher than those in control group (*P* < 0.05). KGF-2 pre-treatment significantly decreased MPO activity compared with the HVZP group (*P* < 0.05). (C) Tumour necrosis factor (TNF)-α in BALF. TNF-α in BALF in HVZP group was significantly higher than those in control group (*P* < 0.05). KGF-2 pre-treatment significantly decreased TNF-α in BALF compared with the HVZP groups (*P* < 0.05). (D) Macrophage inflammatory protein (MIP)-2 level in BALF was significantly higher in HVZP group than those in control group (*P* < 0.05). KGF-2 pre-treatment significantly decreased MIP-2 level in BALF compared with the HVZP group (*P* < 0.05). *P < 0.05 *versus* control group; †*P* < 0.05 *versus*HVZP group.

The number of neutrophil in the BALF and lung MPO activity were significantly increased in rats ventilated with HVZP than other animals (*P* < 0.05, Fig.2A and B). Meanwhile, KGF-2 pre-treatment significantly decreased neutrophil counts and MPO activity (*P* < 0.05, Fig.2A and B). Both TNF-α and MIP-2 in the BALF were significantly increased in HVZP group compared with those in the control group (*P* < 0.05, Fig.2C and D). Keratinocyte growth factor-2 pre-treatment significantly decreased TNF-α and MIP-2 in the BALF compared with those in the HVZP group (*P* < 0.05, Fig.2C and D).

### KGF-2 promotes alveolar type II cells proliferation and surfactant protein mRNA expression

**Figure 3:**
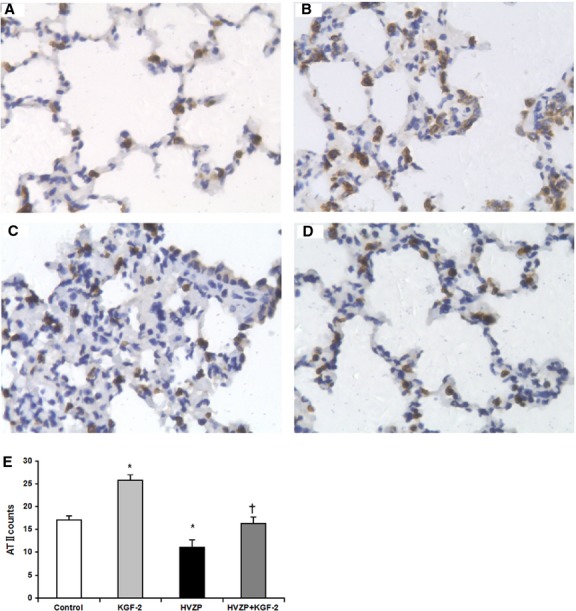
Images of immunohistochemistry for SP-C. (A) control group: Normal rat lung histologic section after intratracheal administration of PBS; (B) KGF-2 group: Marked alveolar type II cell (ATII) hyperplasia is seen after intratracheal administration of KGF-2; (C) HVZP group: alveolar type II cell decreased in lungs subjected to an injurious ventilatory strategy; (D) HVZP+KGF-2 group: KGF-2 pre-treatment increased alveolar type II cell counts for lungs subjected to an injurious ventilatory strategy (×400). (E) Alveolar type II cell counts. Alveolar type II cell count in HVZP group was significantly lower than those in control group (*P* < 0.05). KGF-2 pre-treatment significantly increased alveolar type II cell count compared with the HVZP group (*P* < 0.05). *P < 0.05 *versus* control group; †*P* < 0.05 *versus*HVZP group.

**Figure 4:**
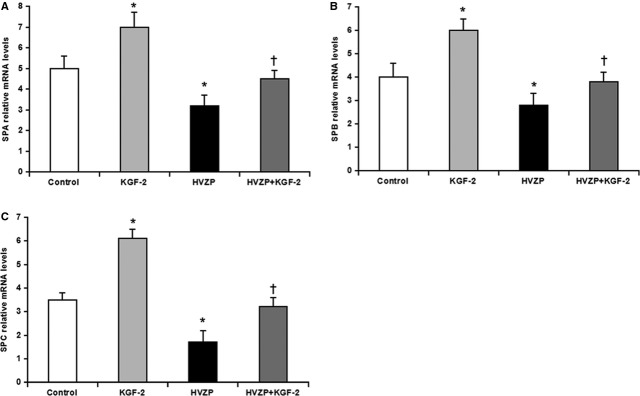
KGF-2 promote SP-A, SB-B and SP-C mRNA expression. (A) SP-A mRNA expression in the lung. SP-A mRNA level was significantly decreased in HVZP group compared with those in control group (*P* < 0.05). KGF-2 pre-treatment significantly increased SP-A mRNA expression compared with the HVZP group (*P* < 0.05). (B) SP-B mRNA expression in the lung. SP-B mRNA level was significantly decreased in HVZP group compared with those in control group (*P* < 0.05). KGF-2 pre-treatment significantly increased SP-B mRNA expression compared with the HVZP group (*P* < 0.05). (C) SP-C mRNA expression in the lung. SP-C mRNA level was significantly decreased in HVZP group compared with those in control group (*P* < 0.05). KGF-2 pre-treatment significantly increased SP-C mRNA expression compared with the HVZP group (*P* < 0.05). *P < 0.05 *versus* control; †*P* < 0.05 *versus*HVZP.

Surfactant protein-C immunoreactivities were mainly detected in alveolar type II cells, and the immunoreactivity markedly decreased in rats treated with HVZP when compared with the control groups (*P* < 0.05, Fig.3). Keratinocyte growth factor-2 pre-treatment significantly increased the number of alveolar type II cells (*P* < 0.05, Fig.3). On the other hand, mRNA expression of SP-A, SP-B and SP-C were significantly lower in HVZP group than control group (*P* < 0.05, Fig.4A–C), but KGF-2 pre-treatment significantly increased the level of SP-A, SP-B and SP-C mRNA expression group (*P* < 0.05, Fig.4A–C).

### KGF-2 preserves the integrity of alveolar-capillary membrane in VILI

**Figure 5:**
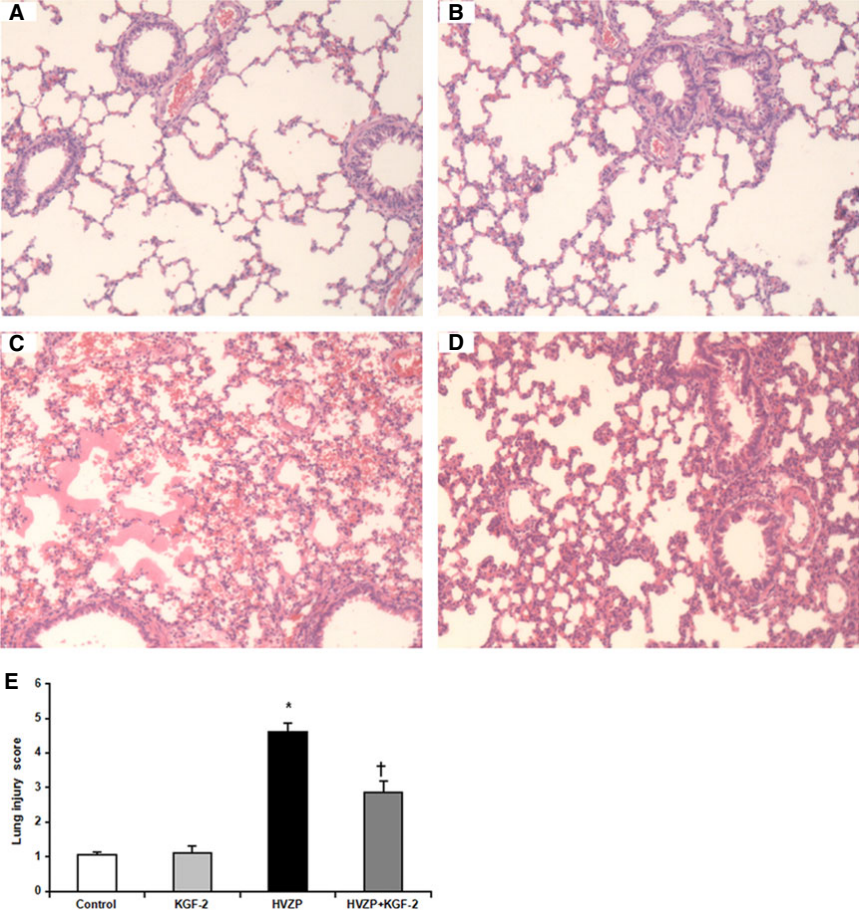
Images of histological specimens under optical microscope stained with haematoxylin and eosin (×100). (A) Control group; (B) KGF-2 group; (C) HVZP group: Widespread alveolar wall thickening, massive neutrophil infiltration, haemorrhage and hyaline membrane formation were observed; (D) HVZP+KGF-2 group: The lung injury was relatively mild. (E) Lung injury score. Score in HVZP group was significantly higher than those in the control group (*P* < 0.05). Compared with HVZP, the score in HVZP+KGF-2 group was significantly decreased (*P* < 0.05). *P < 0.05 *versus* control group; †*P* < 0.05 *versus*HVZP group.

Under optical microscopy, widespread alveolar wall thickening, massive neutrophil infiltration, haemorrhage and hyline membrane forming were observed in HVZP group (Fig.5A–D). The lung injury score in HVZP group was significantly higher than those in other groups (*P* < 0.05, Fig.5E). However, in HVZP+KGF-2 group, the lung injury was mild and the score was significantly lower than that in HVZP group (*P* < 0.05, Fig.5E).

**Figure 6:**
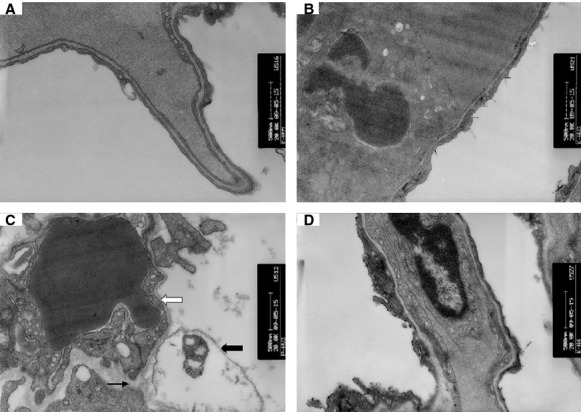
Images of histological specimens from the lungs of animals under transmission electron microscope (×30,000). (A) control group; (B) KGF-2 group; (C) HVZP group: The alveolar–capillary barrier was severely damaged, including alveolar type I cell swelling (thick black arrowhead), basement membrane exposure (thick white arrowhead) and alveolar–capillary barrier rupture (thin black arrowhead). (D) HVZP+KGF-2 group: the alveolar–capillary barrier disruption was relatively mild.

Transmission electron microscopy results revealed that compared with control and KGF-2 groups (Fig.6A and B), the alveolar–capillary barrier was severely damaged in HVZP group, including alveolar type I cell swelling, basement membrane exposure and alveolar–capillary barrier rupture (Fig.6C). In the HVZP+KGF-2 group, the alveolar–capillary barrier disruption was relatively mild (Fig.6D).

## Discussion

The present work confirms that high-volume zero positive end-expiratory pressure ventilation applied for 4 hrs induces lung injury and inflammation in rats. The major finding in the present study was that pre-treatment with KGF-2 could attenuate pulmonary injury in a VILI rat model *in vivo*. The reduction of lung injury with KGF-2 pre-treatment was supported by significantly improved PaO_2_, reduced formation of pulmonary oedema and histological changes. The alveolar-space protein measurements further verified the protective effect of KGF-2, at least in part, by preservation of the alveolar–capillary barrier. Furthermore, KGF-2 pre-treatment could reduce neutrophil infiltration and activation, decrease inflammatory cytokines releasing and preserve alveolar type II cells and surfactant protein expression.

Typical features of VILI are increased alveolar–capillary permeability, cytokine or chemokine production, protein-rich oedema formation and, ultimately, impaired gas exchange [24]. Despite clinical advances in limiting VILI through reduction in V_T_ and application of PEEP [25], these measures may only partly reduce the detrimental effects of MV. Changes in ventilatory strategy can also have limiting negative effects, including impairment of cardiac output with high levels of PEEP, and difficulty in maintaining oxygenation with lower tidal volumes [26]. On the other hand, even a low-tidal volume mode of MV may induce pro-inflammatory response [27] and VILI [28]. Thus, a pharmacological intervention may provide potential new therapies for VILI.

Recently, accumulating evidence has shown that KGF-2, which is critical for lung development and a potent alveolar type II cell mitogen, plays an important role in preventing alveolar epithelial cell DNA damage and apoptosis *via* MAPK/ERK pathway [29,13,12]. Other researchers have reported that pre-treatment with KGF-2 could significantly reduce high-altitude pulmonary oedema in rat model, most likely through down-regulation of apoptosis, and proliferation of type II cells in alveolar space [20]. However, it is unclear whether KFF-2 has the potential to prevent VILI, especially *in vivo*. KGF-2 may affect alveolar–microvascular permeability in a number of ways. As shown by TEM, widespread endothelial and epithelial cell injury was one of the hallmarks of the entity and accounted at least in part for the increased microvascular permeability of ventilator-injured lungs [13]. Several groups have demonstrated that mechanical stretch of cells in culture or high-tidal volume *in vivo* causes alveolar type II cell injury [12,30], apoptosis [31,32], necrosis [33,34], and inhibits wound closure by inhibiting cell spreading and migration [35], which is critical for VILI development [24]. Upadhyay D and coworkers examined the effect of KGF-2 on cyclic mechanical stretch in cultured alveolar type II pneumocytes and they found that KGF-2 could attenuate cyclic stretch-induced alveolar epithelial cell DNA damage *via* MAPK/ERK pathway [29]. Several studies have suggested that inflammatory cells and mediators play important roles in the pathogenesis of VILI [36]. The endothelial cell disruptions during overinflation oedema in small animals allow direct contact between polymorphonuclear cells and basement membrane which promotes leucocyte activation [24]. A striking feature of the VILI that occurs after several hours in larger animals is the infiltration of inflammatory cells into the interstitial and alveolar spaces. In one of the earliest studies on this subject, Woo and Hedley-white [37] observed that overinflation produced oedema in open-chest dogs, and leucocytes accumulated in the vasculature and macrophages in the alveoli. Conversely, when animals were depleted in neutrophils, high-volume pulmonary oedema was less severe than undepleted animals [24], suggesting neutrophils may play important roles in VILI.

Pro-inflammatory cytokines, such as IL-1β and TNF-α, are secreted by alveolar macrophages upon mechanical stretch [38] and they are capable of stimulating endothelial cell activation [39]. In turn, cytokine-activated endothelial cells secrete chemokines and express adhesion molecules on their surface, resulting in enhanced leucocyte adhesiveness and transmigration of activated immune cells across the endothelium of inflamed tissue [40]. Tremblay *et al*. [36] examined the effects of different ventilatory strategies on the expression of several cytokines in BALF of isolated rat lungs ventilated with different end-expiratory pressures and V_T_. Finally, high V_T_ ventilation (40 ml/kg bw) with ZEEP was discovered significantly increased the TNF-α, IL-1ß and IL-6 and in MIP-2 levels (a potent neutrophil chemoattractant and the rodent functional homologue of human IL-8).

Moreover, we also found high V_T_ ventilation (20 ml/kg bw) with ZEEP significantly increased neutrophil counts in BALF and MPO activity in the lung tissue, as well as TNF-α and MIP-2 levels in BALF. However, in the KGF-2 pre-treatment group, the neutrophil counts in BALF, MPO activity in the lung tissue, TNF-α and MIP-2 in BALF were significantly lower than those in HVZP group, which suggested KGF-2 may partly decrease neutrophils infiltration into alveolar spaces, cytokine and chemokine expression, and eventually attenuate the development of an overwhelming inflammatory response.

In addition to its effects on fluid filtration, surfactant inactivation and elevated alveolar surface tension may also increase alveolar epithelial permeability to small solutes. Diethylenetriaminepenta-acetic acid (DTPA) clearance in rabbits [41] and dogs [42] was increased following surfactant inactivation by detergent aerosolization. This effect was ascribed to the uneven distribution of lung mechanical properties resulting in ventilation inhomogeneities and regional overexpansion, rather than to the elimination of peculiar barrier properties of surfactant. The effects of surfactant inactivation and large V_T_ ventilation on alveolocapillar permeability (as assessed by pulmonary DTPA clearance) are additive [43]. Increased surface tension may also alter endothelial permeability as a result of increased radial traction on pulmonary microvessels [38]. The present study showed that, in the normal lung, KGF-2 increased the expression of SP-A, SP-B and SP-C, and HVZP significantly suppressed the expression. However, the levels in HVZP+ KGF-2 group were partly recovered compared with HVZP, which indicated that KGF-2 pre-treatment could prevent the surfactant decrease and then attenuate VILI.

In the present study, KGF-2 has only been effective as a pre-treatment in animal models. In humans, the development of acute lung injury is rarely predictable. Thus, a preventive therapy lacks appeal. The preventive therapy might be useful such as operation patients receiving V_T_ ventilation to reduce the incidence of ALI/ARDS. However, it should be noted that the available animal models of acute lung injury do not adequately reproduce the clinical situation. In humans with acute lung injury, ventilatory and haemodynamic support along with treatment for the underlying inciting clinical disorder may provide a prolonged interval for KGF-2 to exert its therapeutic effects, a situation that cannot be reproduced in animal models. Indeed, the improvement of KGF-2 survival should be further confirmed in our previous study [20], and the detailed potential mechanisms of KGF-2 on VILI are needed to be studied in future.

In conclusion, we demonstrated a protective effect of KGF-2 pre-treatment in a VILI rat model *in vivo*. The protective effect is multifactorial and probably related to improved alveolar–capillary barrier permeability, reduced neutrophil infiltration and activation, decreased inflammatory cytokines releasing and preserved surfactant protein expression. Based on these findings, the delivery of KGF-2 to the lungs may provide a new prophylactic strategy to enhance alveolar epithelial repair during lung damage because of MV or other injurious agents.

## Supporting Information

**Data S1** Preliminary study.

## References

[1] Dreyfuss D, Basset G, Soler P (1985). Intermittent positive-pressure hyperventilation with high inflation pressures produces pulmonary microvascular injury in rats. Am Rev Respir Dis.

[2] Terragni PP, Rosboch G, Tealdi A (2007). Tidal hyperinflation during low tidal volume ventilation in acute respiratory distress syndrome. Am J Respir Crit Care Med.

[3] Dreyfuss D, Saumon G (1998). Ventilator-induced lung injury: lessons from experimental studies. Am J Respir Crit Care Med.

[4] Kim N, Yamamoto H, Pauling MH (2009). Ablation of lung epithelial cells deregulates FGF-10 expression and impairs lung branching morphogenesis. Anat Rec.

[5] Benjamin JT, Smith RJ, Halloran BA (2007). Fgf-10 is decreased in bronchopulmonary dysplasia and suppressed by toll-like receptor activation. Am J Physiol Lung Cell Mol Physiol.

[6] Hyatt BA, Shangguan X, Shannon JM (2004). Fgf-10 induces SPC and bmp4 and regulates proximal-distal patterning in embryonic tracheal epithelium. Am J Physiol Lung Cell Mol Physiol.

[7] Ware LB, Matthay MA (2002). Keratinocyte and hepatocyte growth factors in the lung: roles in lung development, inflammation, and repair. Am J Physiol Lung Cell Mol Physiol.

[8] Tiozzo C, De Langhe S, Carraro G (2009). Fgf10 (fibroblast growth factor 10) plays a causative role in the tracheal cartilage defects in a mouse model of apert syndrome. Pediatr Res.

[9] Clark JC, Tichelaar JW, Wert SE (2001). Fgf-10 disrupts lung morphogenesis and causes pulmonary adenomas *in vivo*. Am J Physiol Lung Cell Mol Physiol.

[10] Sekine K, Ohuchi H, Fujiwara M (1999). Fgf10 is essential for limb and lung formation. Nat Genet.

[11] Gupte VV, Ramasamy SK, Reddy R (2009). Overexpression of fibroblast growth factor-10 during both inflammatory and fibrotic phases attenuates bleomycin-induced pulmonary fibrosis in mice. Am J Respir Crit Care Med.

[12] Upadhyay D, Panduri V, Kamp DW (2005). Fibroblast growth factor-10 prevents asbestos-induced alveolar epithelial cell apoptosis by a mitogen-activated protein kinase-dependent mechanism. Am J Respir Cell Mol Biol.

[13] Upadhyay D, Bundesmann M, Panduri V (2004). Fibroblast growth factor-10 attenuates H_2_O_2_-induced alveolar epithelial cell DNA damage: role of MAPK activation and DNA repair. Am J Respir Cell Mol Biol.

[14] Beer HD, Vindevoghel L, Gait MJ (2000). Fibroblast growth factor (FGF) receptor 1-iiib is a naturally occurring functional receptor for FGFs that is preferentially expressed in the skin and the brain. J Biol Chem.

[15] Igarashi M, Finch PW, Aaronson SA (1998). Characterization of recombinant human fibroblast growth factor (FGF)-10 reveals functional similarities with keratinocyte growth factor (FGF-7). J Biol Chem.

[16] Yamasaki M, Miyake A, Tagashira S (1996). Structure and expression of the rat mRNA encoding a novel member of the fibroblast growth factor family. J Biol Chem.

[17] Emoto H, Tagashira S, Mattei MG (1997). Structure and expression of human fibroblast growth factor-10. J Biol Chem.

[18] Yang J, Meyer M, Muller AK (2010). Fibroblast growth factor receptors 1 and 2 in keratinocytes control the epidermal barrier and cutaneous homeostasis. J Cell Biol.

[19] Nakamura T, Mochizuki Y, Kanetake H (2001). Signals *via* FGF receptor 2 regulate migration of endothelial cells. Biochem Biophys Res Commun.

[20] She J, Goolaerts A, Shen J (2012). KGF-2 targets alveolar epithelia and capillary endothelia to reduce high altitude pulmonary oedema in rats. J Cell Mol Med.

[21] Freytes CO, Ratanatharathorn V, Taylor C (2004). Phase I/II randomized trial evaluating the safety and clinical effects of repifermin administered to reduce mucositis in patients undergoing autologous hematopoietic stem cell transplantation. Clin Cancer Res.

[22] Choi WI, Quinn DA, Park KM (2003). Systemic microvascular leak in an *in vivo* rat model of ventilator-induced lung injury. Am J Respir Crit Care Med.

[23] Johansson J, Curstedt T, Robertson B (1994). The proteins of the surfactant system. Eur Respir J.

[24] Ricard JD, Dreyfuss D, Saumon G (2003). Ventilator-induced lung injury. Eur Respir J Suppl.

[25] Parker JC, Hernandez LA, Peey KJ (1993). Mechanisms of ventilator-induced lung injury. Crit Care Med.

[26] Welsh D, Summer W, de Boisblanc B (1999). Hemodynamic consequences of mechanical ventilation. Clin Pulm Med.

[27] Caruso P, Meireles SI, Reis LF (2003). Low tidal volume ventilation induces proinflammatory and profibrogenic response in lungs of rats. Intensive Care Med.

[28] Wolthuis EK, Vlaar AP, Choi G (2009). Mechanical ventilation using non-injurious ventilation settings causes lung injury in the absence of pre-existing lung injury in healthy mice. Crit Care.

[29] Upadhyay D, Meyer EC, Sznajder JI (2003). FGF-10 attenuates cyclic stretch-induced alveolar epithelial cell DNAdamage *via* MAPK/ERK pathway. Am J Physiol Lung Cell Mol Physiol.

[30] Vlahakis NE, Schroeder MA, Pagano RE (2002). Role of deformation-induced lipid trafficking in the prevention of plasma membrane stress failure. Am J Respir Crit Care Med.

[31] Hammerschmidt S, Kuhn H, Gessner C (2007). Stretch-induced alveolar type II cell apoptosis: role of endogenous bradykinin and PI3K-Akt signaling. Am J Respir Cell Mol Biol.

[32] Hammerschmidt S, Kuhn H, Grasenack T (2004). Apoptosis and necrosis induced by cyclic mechanical stretching in alveolar type II cells. Am J Respir Cell Mol Biol.

[33] Cavanaugh KJ, Margulies SS (2002). Measurement of stretch-induced loss of alveolar epithelial barrier integrity with a novel *in vitro* method. Am J Physiol Cell Physiol.

[34] Tschumperlin DJ, Oswari J, Margulies AS (2000). Deformation-induced injury of alveolar epithelial cells. Effect of frequency, duration, and amplitude. Am J Respir Crit Care Med.

[35] Savla U, Waters CM (1998). Mechanical strain inhibits repair of airway epithelium *in vitro*. Am J Physiol Lung Cell Mol Physiol.

[36] Tremblay L, Valenza F, Ribeiro SP (1997). Injurious ventilatory strategies increase cytokines and c-fos mRNA expression in an isolated rat lung model. J Clin Invest.

[37] Woo SW, Hedley-White J (1972). Macrophage accumulation and pulmonary edema due to thoracotomy and lung overinflation. J Appl Physiol.

[38] Pugin J, Dunn I, Jolliet P (1998). Activation of human macrophages by mechanical ventilation *in vitro*. Am J Physiol.

[39] Meager A (1999). Cytokine regulation of cellular adhesion molecule expression in inflammation. Cytokine Growth Factor Rev.

[40] Hegeman MA, Hennus MP, Heijnen CJ (2009). Ventilator-induced endothelial activation and inflammation in the lung and distal organs. Crit Care.

[41] Jefferies AL, Kawano T, Mori S (1988). Effect of increased surface tension and assisted ventilation on 99mTc-DTPA clearance. J Appl Physiol.

[42] Nieman G, Ritter-Hrncirik C, Grossman Z (1990). High alveolar surface tension increases clearance of technetium 99m diethylenetriaminepentaacetic acid. J Thorac Cardiovasc Surg.

[43] John J, Taskar V, Evander E (1997). Additive nature of distension and surfactant perturbation on alveolocapillary permeability. Eur Respir J.

