# Supplementary material for: Keratinocyte growth factor-2 intratracheal instillation significantly attenuates ventilator-induced lung injury in rats
Source: J Cell Mol Med. 2014 Mar 21;18(6):1226–35. doi: 10.1111/jcmm.12269 (PMC4508161; doi:10.1111/jcmm.12269)

**Supporting Information**

**Keratinocyte Growth Factor-2 intra-tracheal instillation significantly attenuates Ventilator-induced Lung Injury in Rats**

Jing Bi M.S.^#^, Lin Tong M.S.^#^, Xiaodan Zhu M.D., Dong Yang M.D.,Ph.D., Chunxue Bai M.D.,Ph.D.*, Yuanlin Song M.D. ^*^ and Jun She M.D.,Ph.D.^*^

Department of Pulmonary Medicine, Zhongshan Hospital, Fudan University, Shanghai, China.

^#^ These authors contribute to this work equally

^*^Corresponding Author:

Jun She M.D.,PhD

Address: 180 Fenglin Road, Shanghai, China, 200032

Phone: +86(021)64041990-2445

E-mail: shejuncn@aliyun.com

Yuanlin Song, M.D.

Address: 180 Fenglin Road, Shanghai, China, 200032

Phone: +86(021)64041990-2445

Email: [ylsong70@gmail.com](mailto:ylsong70@gmail.com)

Chunxue Bai M.D.,Ph.D.

Address: 180 Fenglin Road, Shanghai, China, 200032

Phone: +86(021)64041990-3077

Email: bai.chunxue@zs-hospital.sh.cn

**Preliminary study**

**Objective**

To determine the optimal dose and administration time of KGF-2. To determine whether the preventive effect of KGF-2 on VILI was dose or time dependent.

**Methods**

***Animals***

Healthy Specific pathogen Free Male Sprague-Dawley (SD) rats (the Animal Center of Fudan University, Shanghai, China) weighing 200–250 g were used. Rats were maintained in the laboratory animal center of Zhongshan Hospital, Fudan University, Shanghai with clean, controlled temperature and independent ventilation environment. The animals had free access to food and water, but food was withdrawn 12 hours before experiment. All procedures were approved by the Committee of Animal Care of Fudan University. All animals were handled in accordance with the Guide for the Care and Use of Laboratory Animals published by the National Institutes of Health, and efforts were made to minimize suffering.

***Experimental design***

There were three groups of animals(N=8): 1) an untreated group of controls, 2) a vehicle pre-treated group, and 3) a KGF-2 pre-treated group. The untreated controls were neither anesthetized nor instilled. The vehicle-

treated group received PBS (0.5 ml) as the vehicle for KGF-2. The KGF-2-treated group was given KGF (1, 2, 5, 10 mg/kg in 0.5 ml of PBS). The day of PBS or KGF-2 instillation was designated day 0. Animals in the vehicle-treated group and KGF-2-treated group were underwent high-volume zero positive end-expiratory pressure (PEEP) (HVZP)ventilation strategy for 4 hours 1, 2, 3, 5, and 7 days after instillation.

***Animal model of Ventilator induced lung injury***

The protocol was described in manuscript.

***Measurement of HVZP-induced hypoxemia and protein rich oedema***

At the end of each experiment,arterial blood gas, lung W/D Weight Ratio, BAL cell count and BAL protein concentration were measured to evaluate HVZP-induced hypoxemia and protein rich oedema. The details were described in manuscript.

***Statistical Analysis***

Data were presented as mean ± SD (standard deviation) and analyzed using SPSS version 11.5 statistical software (SPSS, Chicago, IL). Comparisons between multiple groups were performed by one-way ANOVA procedures, followed by the Bonferroni post hoc test for intergroup comparisons. The histologic semiquantitative analysis was compared by the nonparametric Mann-Whitney test. A p value ＜ 0.05 was considered significant.

**Results**

**KGF-2 prevented HVZP-induced hypoxemia and protein rich oedema dose-dependently**

In order to determine the optimal dose of KGF-2, rats were pre-treated with PBS or KGF-2(1, 2, 5, 10mg/kg) three days before HVZP ventilation. After 4h HVZP ventilation, the partial pressure of oxygen (PaO2) of rats in PBS pre-treated group was significantly lower than that in untreated control group (p < 0.05, supFig. 1A). KGF-2(2, 5, 10mg/kg) pretreated groups signficantly increased PaO2 compared with the PBS pre-treated groups (p < 0.05, Fig. 1A). In addition, the PaO2 of KGF-2(5mg/kg) pre-treated group was significantly higher than that in KGF-2(1mg/kg) pre-treated group and KGF-2(2mg/kg) pre-treated group (p < 0.05, supFig. 1A), but values were similar between those pre-treated with KGF-2(5mg/kg) and KGF-2(10mg/kg).

Lung W/D Weight Ratio, BAL cell count and BAL protein concentration were measured to evaluate HVZP-induced protein rich oedema. The result suggested that

lung W/D Ratio, BAL cell count and BAL protein concentration in PBS pre-treated group were significantly increased than those in untreated control group (p < 0.05, supFig. 1B,1C,1D). KGF-2(2, 5, 10mg/kg) pre-treated groups signficantly decreased lung W/D Ratio, BAL cell count and BAL protein concentration compared with the PBS pre-treated groups (p < 0.05, supFig. 1B,1C,1D). In addition, those values of KGF-2(5mg/kg) pre-treated group were significantly lower than those in KGF-2(1mg/kg) pre-treated group and KGF-2(2mg/kg) pre-treated group (p < 0.05, supFig.1B,1C,1D), but values were similar between those pre-treated with KGF-2(5mg/kg) and KGF-2(10mg/kg).

The results mentioned above indicated that KGF-2 prevented HVZP-induced hypoxemia and protein rich oedema dose-dependently, KGF-2 could provide maximum protection with 5 mg/kg.

**KGF-2 pre-treated 3days before HVZP ventilation providing maximum protection**

In order to determine the optimal administration time of KGF-2, rats were pre-treated with PBS or KGF-2 (5mg/kg) 1, 2, 3, 5, and 7 days before HVZP ventilation. After 4h HVZP ventilation, the partial pressure of oxygen (PaO2) of rats in PBS pre-treated group was significantly lower than that in untreated control group (p < 0.05, supFig. 2A). KGF-2(5mg/kg) 2, 3, 5days pretreated groups signficantly increased PaO2 compared with the PBS pre-treated group (p < 0.05, Fig. 2A). In addition, the PaO2 of KGF-2(5mg/kg) 3 days pre-treated group was significantly higher than that in KGF-2(5mg/kg) 1, 2, 5, 7 days pre-treated groups(p < 0.05, supFig. 2A).

Lung W/D Weight Ratio, BAL cell count and BAL protein concentration were measured to evaluate HVZP-induced protein rich oedema. The result suggested that

lung W/D Ratio, BAL cell count and BAL protein concentration in PBS pre-treated group were significantly increased than those in untreated control group (p < 0.05, supFig. 2B,2C,2D). KGF-2(5mg/kg) 2, 3, 5days pre-treated groups signficantly decreased lung W/D Ratio, BAL cell count and BAL protein concentration compared with the PBS pre-treated groups (p < 0.05, supFig. 2B,2C,2D). In addition, those values of KGF-2(5mg/kg) 3 days pre-treated group were significantly lower than those in KGF-2(5mg/kg) 1, 2, 5, 7 days pre-treated groups (p < 0.05, supFig. 2B,2C,2D).

The results mentioned above indicated that KGF-2 could provide maximum protection with 5 mg/kg 3days before ventilation.

**Figure legends**

**supFigure 1. KGF-2 improves HVZP-induced hypoxemia and protein rich oedema dose-dependently**

**supFigure 1A:** partial pressure of oxygen (PaO_2_.).**supFigure 1B:** lung W/D weight ratios. **supFigure 1C:** The number of total cells recovered in the bronchoalveolar lavage fluid (BALF). **supFigure 1D:** The total protein contents recovered in the bronchoalveolar lavage fluid. Rats were pre-treated with PBS or KGF-2(1, 2, 5, 10mg/kg) three days before HVZP ventilation.After 4h HVZP ventilation, the partial pressure of oxygen (PaO2) of rats in PBS pre-treated group was significantly lower than that in untreated control group (p < 0.05). KGF-2(2, 5, 10mg/kg) pretreated groups signficantly increased PaO2 compared with the PBS pre-treated groups (p < 0.05). In addition, the PaO2 of KGF-2(5mg/kg) pre-treated group was significantly higher than that in KGF-2(1mg/kg) pre-treated group and KGF-2(2mg/kg) pre-treated group (p < 0.05), but values were similar between those pre-treated with KGF-2(5mg/kg) and KGF-2(10mg/kg). Lung W/D Ratio, BAL cell count and BAL protein concentration in PBS pre-treated group were significantly increased than those in untreated control group (p < 0.05). KGF-2(2, 5, 10mg/kg) pre-treated groups signficantly decreased lung W/D Ratio, BAL cell count and BAL protein concentration compared with the PBS pre-treated groups (p < 0.05). In addition, those values of KGF-2(5mg/kg) pre-treated group were significantly lower than those in KGF-2(1mg/kg) pre-treated group and KGF-2(2mg/kg) pre-treated group (p < 0.05), but values were similar between those pre-treated with KGF-2(5mg/kg) and KGF-2(10mg/kg).

# *p* < 0.05 versus untreated control group; † *p* < 0.05 versus PBS pre-treated group;* *p* < 0.05 versus KGF-2(5mg/kg) pre-treated group.

**supFigure 2. KGF-2 pre-treated 3days before HVZP ventilation providing maximum protection**

**supFigure 2A:** partial pressure of oxygen (PaO_2_.).**supFigure 2B:** lung W/D weight ratios. **supFigure 2C:** The number of total cells recovered in the bronchoalveolar lavage fluid (BALF). **supFigure 2D:** The total protein contents recovered in the bronchoalveolar lavage fluid. Rats were pre-treated with PBS or KGF-2 (5mg/kg) 1, 2, 3, 5, and 7 days before HVZP ventilation. After 4h HVZP ventilation, the partial pressure of oxygen (PaO2) of rats in PBS pre-treated group was significantly lower than that in untreated control group (p < 0.05). KGF-2(5mg/kg) 2, 3, 5days pretreated groups signficantly increased PaO2 compared with the PBS pre-treated group (p < 0.05). In addition, the PaO2 of KGF-2(5mg/kg) 3 days pre-treated group was significantly higher than that in KGF-2(5mg/kg) 1, 2, 5, 7 days pre-treated groups(p < 0.05). Lung W/D Ratio, BAL cell count and BAL protein concentration in PBS pre-treated group were significantly increased than those in untreated control group (p < 0.05). KGF-2(5mg/kg) 2, 3, 5days pre-treated groups signficantly decreased lung W/D Ratio, BAL cell count and BAL protein concentration compared with the PBS pre-treated groups (p < 0.05). In addition, those values of KGF-2(5mg/kg) 3 days pre-treated group were significantly lower than those in KGF-2(5mg/kg) 1, 2, 5, 7 days pre-treated groups (p < 0.05). # *p* < 0.05 versus untreated control group; †*p* < 0.05 versus PBS pre-treated group; ** p* < 0.05 versus KGF-2(5mg/kg) 3days pre-treated group.

**supFigure 1**


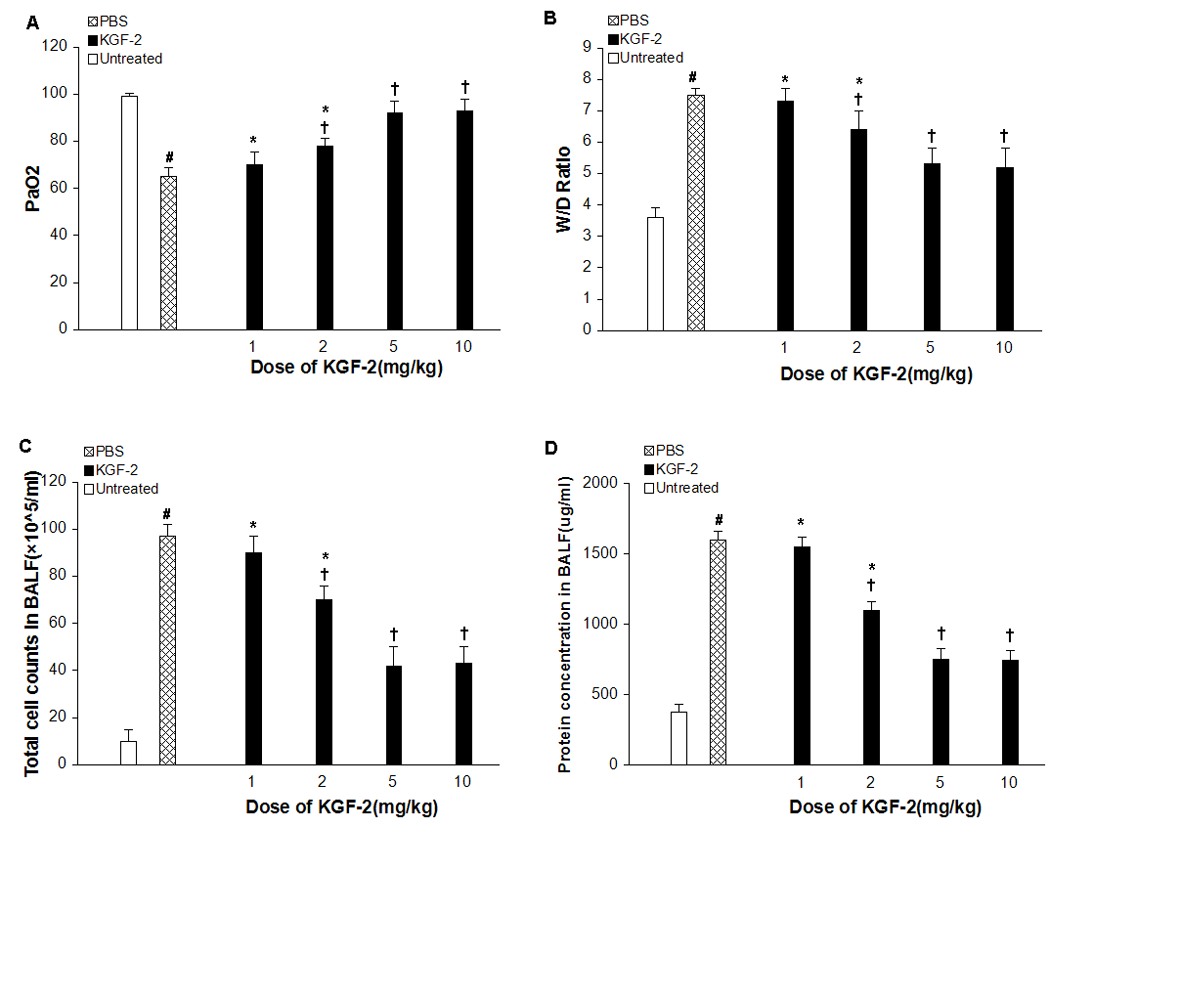


**supFigure 2**


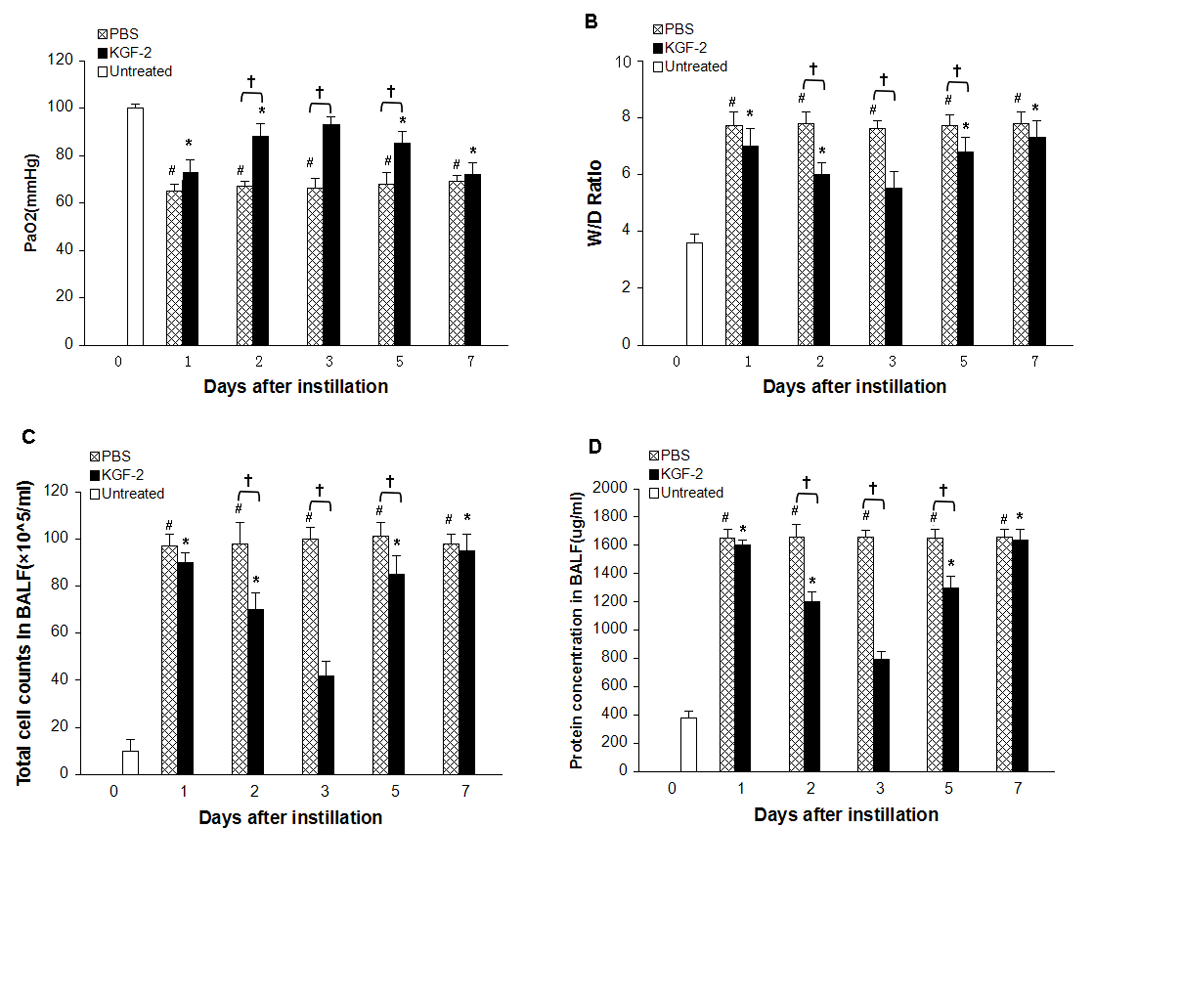

Supplement: Supplementary file 1 [file jcmm0018-1226-sd1.doc]
